# Nonintubated Thoracoscopic Lobectomy for Lung Cancer Using Epidural Anesthesia and Intercostal Blockade: A Retrospective Cohort Study of 238 Cases

**DOI:** 10.1097/MD.0000000000000727

**Published:** 2015-04-03

**Authors:** Ming-Hui Hung, Kuang-Cheng Chan, Ying-Ju Liu, Hsao-Hsun Hsu, Ke-Cheng Chen, Ya-Jung Cheng, Jin-Shing Chen

**Affiliations:** From the Department of Anesthesiology (MHH, KCC, YJL, YJC); Graduate Institute of Clinical Medicine (MHH, KCC); Division of Thoracic Surgery (HHH, KCC, JSC), Department of Surgery; and Department of Traumatology (JSC), National Taiwan University Hospital and National Taiwan University College of Medicine, Taipei, Taiwan.

## Abstract

Intubated general anesthesia with single-lung ventilation has been considered mandatory for thoracoscopic lobectomy for nonsmall cell lung cancer. Few reports of thoracoscopic lobectomy without tracheal intubation are published, using either thoracic epidural anesthesia (TEA) or intercostal blockade. The comparisons of perioperative outcomes of nonintubated thoracoscopic lobectomy using epidural anesthesia and intercostal blockade are not reported previously.

From September 2009 to August 2014, a total of 238 patients with lung cancer who underwent nonintubated thoracoscopic lobectomy were recruited from our prospectively maintained database of all patients undergoing nonintubated thoracoscopic surgery using TEA or intercostal blockade. A multiple regression analysis, adjusting for preoperative variables, was performed to compare the perioperative outcomes of the 2 anesthesia methods.

Overall, 130 patients underwent nonintubated thoracoscopic lobectomy using epidural anesthesia whereas 108 had intercostal blockade. The 2 groups were similar in demographic data, except for sex, preoperative lung function, physical status classification, and history of smoking. After adjustment for the preoperative variables, nonintubated thoracoscopic lobectomy using intercostal blockade was associated with shorter durations of anesthetic induction and surgery (*P* < 0.001). Furthermore, hemodynamics were more stable with less use of vasoactive drugs (odds ratio: 0.53; 95% confidence interval [CI], 0.27 to 1.04; *P* = 0.064) and less blood loss (mean difference: −55.2 mL; 95% CI, −93 to −17.3; *P* = 0.004). Postoperatively, the 2 groups had comparable incidences of complications. Patients in the intercostal blockade group had a shorter average duration of chest tube drainage (*P* = 0.064) but a similar average length of hospital stay (*P* = 0.569). Conversion to tracheal intubation was required in 13 patients (5.5%), and no in-hospital mortality occurred in either group.

Nonintubated thoracoscopic lobectomy using either epidural anesthesia or intercostal blockade is feasible and safe. Intercostal blockade is a simpler alternative to epidural anesthesia for nonintubated thoracoscopic lobectomy in selected patients with lung cancer.

## INTRODUCTION

Lung cancer is still the leading cause of cancer deaths in Taiwan and worldwide.^1^ Among all lung cancers, >80% are nonsmall cell lung cancer (NSCLC). Currently, a thoracoscopic lobectomy remains the best treatment option for patients with early-stage NSCLC.^2^

Video-assisted thoracoscopic surgery (VATS) is traditionally performed on intubated patients under general anesthesia. Either a double-lumen tube or a bronchial blocker is regarded as mandatory to achieve one-lung ventilation (OLV) and establish a safe operating environment.^3^ Although complications after general anesthesia via intubation are well tolerated by most patients, life-threatening events do occur, and they are not negligible.^4^ Therefore, thoracic surgeries without tracheal intubation have evolved in the past decade using thoracic epidural anesthesia (TEA) with or without conscious sedation.^5–15^

Previous studies have demonstrated that thoracoscopic lobectomy without tracheal intubation in patients with lung cancer is technically feasible and as safe as lobectomy performed with tracheal intubation.^10,11^ In addition, nonintubated thoracoscopic lobectomy offers several advantages over the procedure with tracheal intubation. The benefits include lower rates of sore throat and stridor, earlier resumption of oral intake, fewer complications, and shorter postoperative hospital stays.^10,11^ Nonetheless, the technique of TEA is not only time consuming and requires great skill but can also cause unwanted neurological side effects. Although these side effects are rare, they are devastating if they occur.^16^ Recently, we assessed a modified nonintubated technique using internal intercostal nerve block (INB) that does not require TEA.^17^ It is technically feasible for thoracoscopic lobectomy although its safety and efficacy are unclear. Thus, we compared the safety and short-term outcomes of thoracoscopic lobectomy without intubation using regional anesthesia consisting of either TEA or INB in patients with lung cancer. We hypothesized that for nonintubated thoracoscopic lobectomy, using INB would promptly induce anesthesia, avoid TEA-related complications, and result in better clinical outcomes. Therefore, we conducted a retrospective study to determine the safety and short-term outcomes of nonintubated thoracoscopic lobectomy using regional anesthesia (INB vs TEA) in patients with NSCLC.

## PATIENTS AND METHODS

This study was approved by the Research Ethics Committee of National Taiwan University Hospital, Taipei, Taiwan (201304050RINC). All patients gave their consent before the operation after receiving an explanation of the type of anesthesia and surgical procedure.

### Study Design and Patients

We began performing nonintubated VATS for diagnosis and treatment of lung tumors in August 2009. Therefore, our prospectively maintained database of all patients undergoing nonintubated VATS was retrospectively used to identify patients with lung cancer who underwent thoracoscopic lobectomy for lung cancer. Our thoracic surgical team, comprising surgeons and anesthesiologists, selected appropriate patients for nonintubated VATS after a review of their medical records and the results of physical examinations. The criteria for patients undergoing nonintubated thoracoscopic lobectomy included clinical stage I or II peripheral NSCLC, tumors <6 cm in diameter, and no evidence of involvement of chest wall, diaphragm, or main bronchus.^10–13,17^ Additionally, patients with an American Society of Anesthesiologists (ASA) score of ≥4, a history of bleeding disorder, sleep apnea, previous ipsilateral thoracic surgery, or evidence of pleural adhesions, and patients with unfavorable airways or spinal anatomy, or chest wall deformity were excluded. After explaining the type of anesthesia and surgical procedure, approval for nonintubated VATS was obtained from the patients.

### Anesthetic Setting, Induction, and Maintenance

Our anesthesia protocol for nonintubated VATS is fully described in the previous studies.^10–13,17^ In summary, patients were premedicated with intravenous fentanyl (50–100 μg). Standard monitoring included electrocardiography, pulse oximetry, arterial blood pressure, and respiratory rate. The end-tidal carbon dioxide was measured by insertion of a detector into one nostril.^10^ To monitor the depth of anesthesia, a bispectral index (BIS) sensor (BIS Quatro; Aspect Medical System, Norwood, MA) was routinely applied to the forehead of each patient since 2012.^17^

For patients undergoing TEA, an epidural catheter was first inserted into the T5/T6 thoracic interspace to achieve and maintain a sensory block between the T2 and T9 dermatomes using 2% lidocaine. Patients were sedated with intravenous propofol using a target-controlled infusion method. The sedation level desired was a Ramsay sedation score of III (responding to commands only)^18^ before the use of BIS monitoring and a BIS value between 40 and 60 after application of BIS monitoring.^17^ In addition, incremental intravenous injection of fentanyl 25 μg was given to maintain a respiration rate between 12 and 20 breaths/min.

**FIGURE 1:**
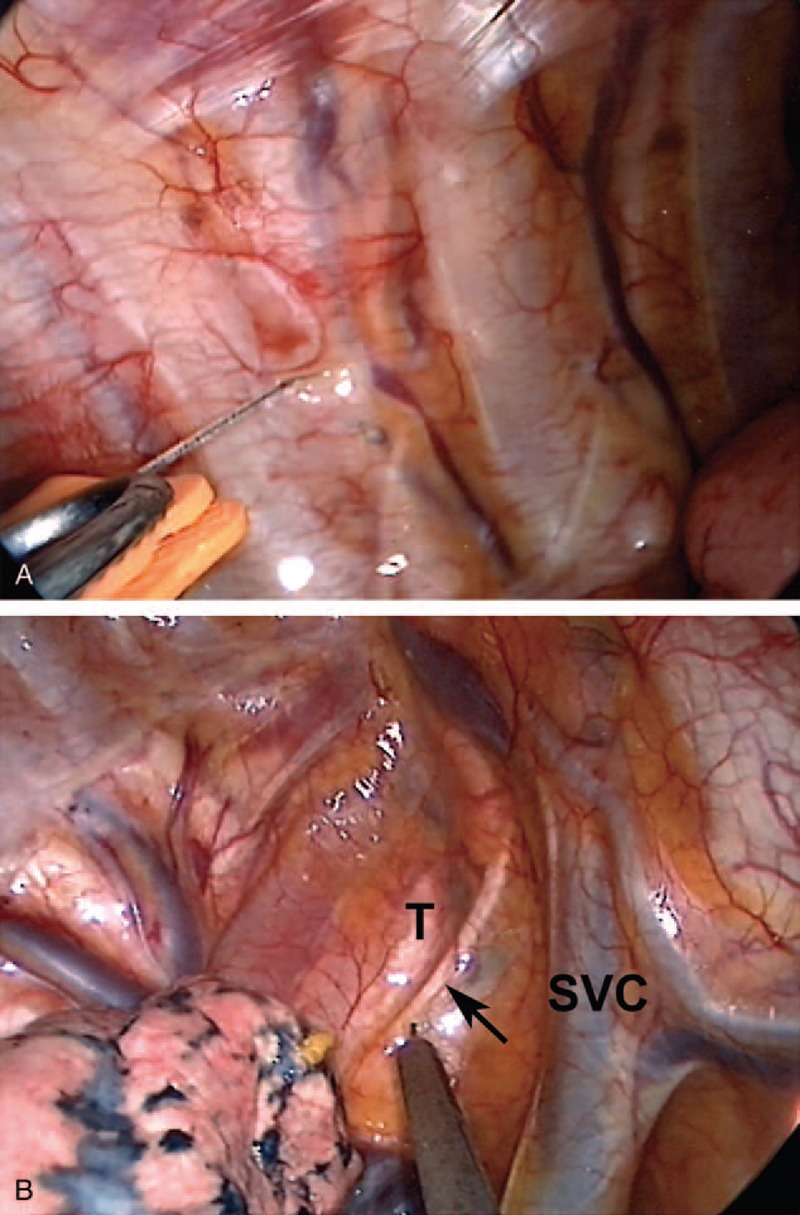
Local infiltration of bupivacaine to produce (A) intercostal blocks with a 25-gauge top-winged infusion needle and (B) intrathoracic vagal block with a long-needle instrument in a representative patient undergoing right-sided nonintubated thoracoscopic lobectomy. The right-sided vagus nerve (arrow) runs between the superior vena cava (SVC) and the lower trachea (T).

For patients undergoing INB, a thoracoscopy port in the midaxillary line and a working port in the auscultatory triangle were initially created after local infiltration with 2% lidocaine. After collapse of the lung undergoing lobectomy, INBs were then produced using direct thoracoscopic vision by infiltration of 0.5% bupivacaine (1.5 mL for each intercostal space) from the third to the eighth intercostal nerves by instillation with a 25-gauge, top-winged infusion needle under the parietal pleura, 2 cm lateral to the sympathetic chain (Figure 1A).^17^

To prevent coughing during thoracoscopic manipulation, an intrathoracic vagal block was produced in all patients. Bupivacaine (2–3 mL) was instilled adjacent to the vagus nerve at the level of the lower trachea for right-sided procedures and at the level of the aortopulmonary window for left-sided procedures (Figure 1B).^10–13,17^ After chest tube insertion, the operated lung was manually expanded via positive-pressure mask ventilation to check for the presence of air leaks. Then, the propofol infusion was stopped. At the end of surgery, patients were fully awake and asked to breathe deeply and cough to reexpand the collapsed lung further.

### Surgical Technique for Thoracoscopic Lobectomy

The surgical protocol for thoracoscopic lobectomy, using the 3-port method of McKenna,^19^ has been described in detail elsewhere.^10,11^ Briefly, the patient was placed in the full lateral decubitus position, with slight flexion of the table at the level of the mid chest. The thoracoscope was placed into the seventh or eighth intercostal space along the midaxillary line. A working port was inserted in the sixth or seventh intercostal space in an auscultatory triangle and an anterior 3 to 5-cm utility incision was placed anteriorly in the fifth intercostal space.

After collapse of the lung, incomplete fissures, pulmonary arteries, veins, and bronchi were divided and sectioned with endoscopic stapling devices. The resected lung lobe was removed in an organ retrieval bag through the utility incision. The rough pleural surface of the lung was repaired using 4-0 prolene after staging dissection of mediastinal lymph nodes. A 28-French chest tube was placed through the lowest incision. Rib spreading, rib cutting, and retractor use were avoided in all patients, except when conversion to thoracotomy was required.

### Conversion to General Anesthesia With Intubation

The attending surgeon and anesthesiologist decided whether or not to convert regional anesthesia to general anesthesia via intubation and OLV in cases of ineffective epidural or intercostal analgesia, persistent hypoxemia (oxygen saturation by pulse oximetry <80%), massive pleural adhesion, unstable hemodynamic status, or intraoperative bleeding requiring a thoracotomy.^10–13,17^ For conversion, the surgical wounds were sealed with transparent waterproof dressings (Tegaderm Film; 3M Health Care, Neuss, Germany) after placement of a chest tube to reexpand the collapsed lung. The trachea was then intubated under the guidance of a bronchoscope, followed by insertion of a bronchial blocker for OLV without changing the patient's position.

### Postoperative Analgesics and Care

Postoperative analgesia was administered either by continuous epidural infusion of bupivacaine 0.1% and fentanyl (1.25 μg/mL) or by patient-controlled analgesia with intravenous morphine (1 mg/mL) for 2 to 3 days. Patients who refused patient-controlled analgesia were given intramuscular morphine 5 mg on demand every 4 to 6 hours. Additional nonsteroidal analgesics were given once patients resumed oral intake 2 to 4 hours after surgery. Chest radiography was performed immediately after surgery or on the next morning. The chest tube was removed if no air leak was present and drainage was <200 mL in a 24-hour period. All postoperative complications requiring medication or intervention were recorded.

### Outcome Variables and Statistical Analyses

The primary outcome variables studied were mortality, conversion to intubation, complications, and duration of postoperative chest drainage and hospital stay for each patient who underwent thoracoscopic lobectomy without intubation. Secondary outcomes included the length of anesthesia induction and surgery, intraoperative cardiopulmonary stability (intraoperative oxygenation and carbon dioxide in the blood during one-lung breathing, frequencies of hypotension, and volume of fluid administered), blood loss, and the number of dissected lymph nodes.

The data are summarized as means (and standard deviations) or medians (ranges) for continuous variables and frequencies (%) for categorical variables. For the statistical analyses, Student *t* test was used for continuous variables and the χ^2^ test or Fisher exact test for categorical variables. Furthermore, multiple regression analyses were performed for adjusting preoperative variables between groups. All analyses were performed using SigmaPlot 12 for Windows (SAS Institute, Cary, NC). Statistical analyses were considered significant at a *P* value <0.05.

## RESULTS

**FIGURE 2:**
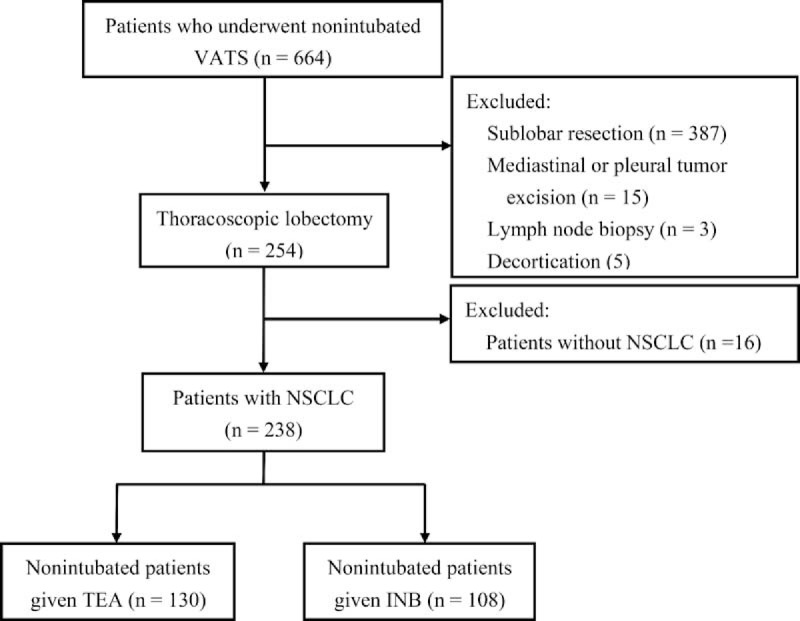
Flow chart of patients screened during the study period. INB = intercostal nerve block, NSCLC = nonsmall cell lung cancer, TEA = thoracic epidural anesthesia, VATS = video-assisted thoracoscopic surgery.

The algorithm for patient selection is shown in Figure 2. From August 2009 through August 2014, VATS without tracheal intubation was performed in 664 patients. Among them, 238 patients with NSCLC underwent lobectomy and mediastinal lymph node dissection, including 130 patients using TEA and 108 using INB (Table 1). The TEA and INB groups did not differ in age, height, weight, body mass index (BMI), tumor location and size, and numbers or types of comorbidities. However, the 2 groups differed in sex, history of smoking, preoperative spirometric lung function, and ASA physical status classification.

**TABLE 1:**
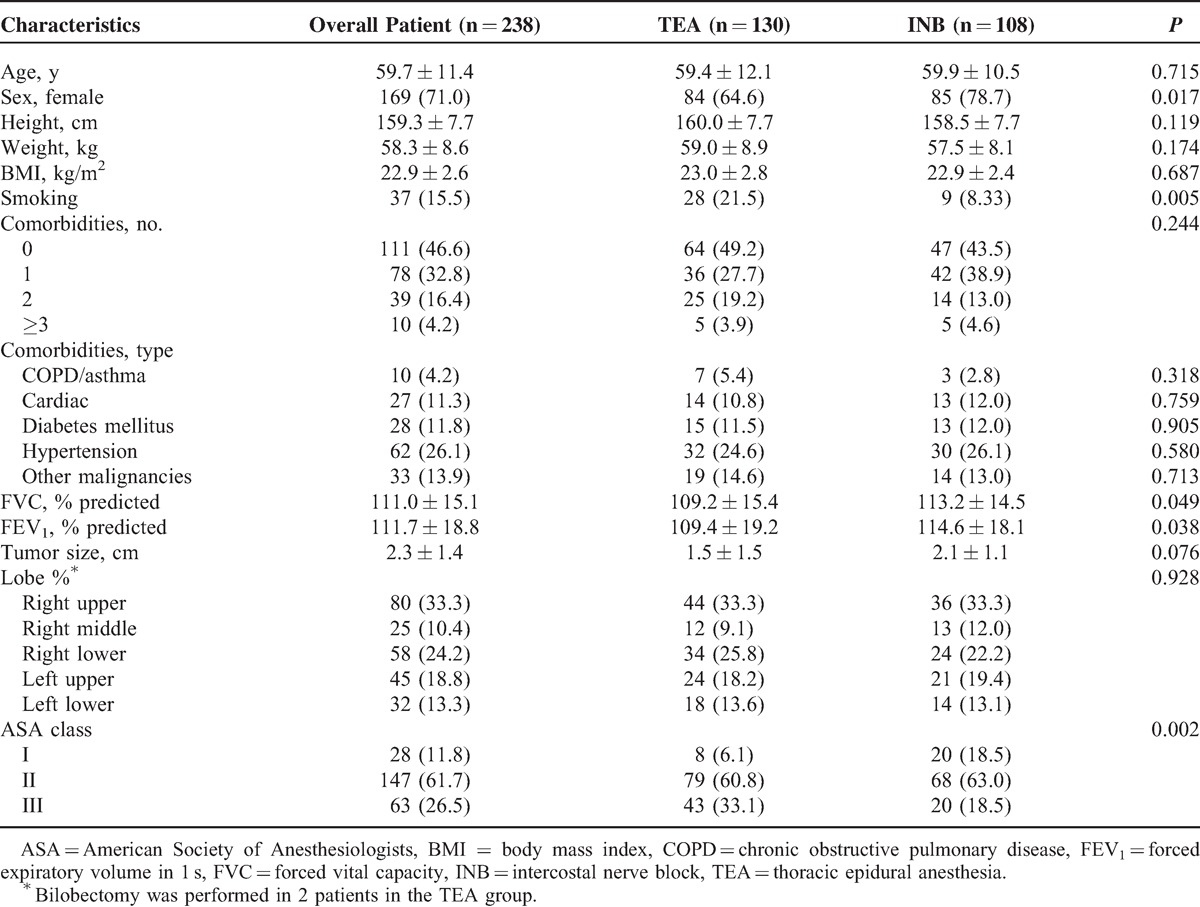
Clinical Characteristics of Nonintubated Patients Undergoing TEA or INB

### Anesthetic and Surgical Results

Patients receiving INB had shorter anesthetic induction time and surgical duration (Table 2). With spontaneous breathing and vagal blockade, collapse of the operative lung and inhibition of coughing were satisfactory in most of the nonintubated patients. The mean detectable lowest oxyhemoglobin saturation by pulse oximetry and highest partial pressure of arterial carbon dioxide during one-lung breathing did not differ significantly between the 2 groups. Oxyhemoglobin saturation and arterial carbon dioxide during one-lung breathing were clinically acceptable and safe in all patients (Table 2). Nonetheless, 13 patients (11 patients given TEA and 2 patients given INB) required conversion to intubation and general anesthesia (Table 3). The reasons for conversion were vigorous mediastinal movement (6 patients) jeopardizing the safety of the surgical environment, bleeding due to vessel injury (3 patients), ineffective TEA (2 patients), dense hilar adhesions (1 patient), and persistent arterial oxygen desaturation (1 patient) (Table 3). Additionally, 1 patient given TEA had a laceration of the pulmonary artery requiring immediate conversion to a thoracotomy to stop the bleeding. After conversion and mechanical ventilation, all operations were smoothly performed.

**TABLE 2:**
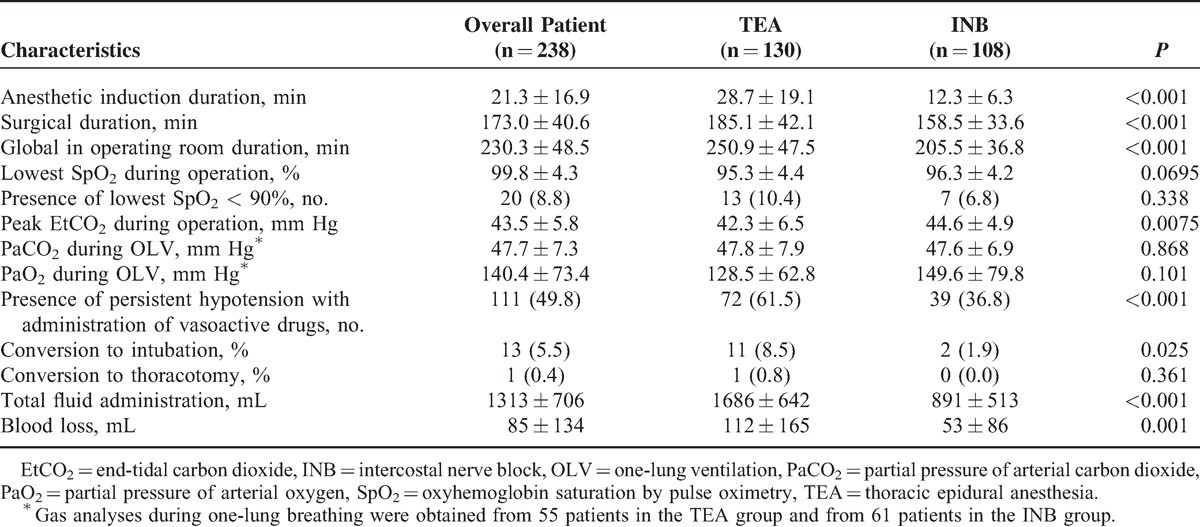
Operative and Anesthetic Results by Group

**TABLE 3:**
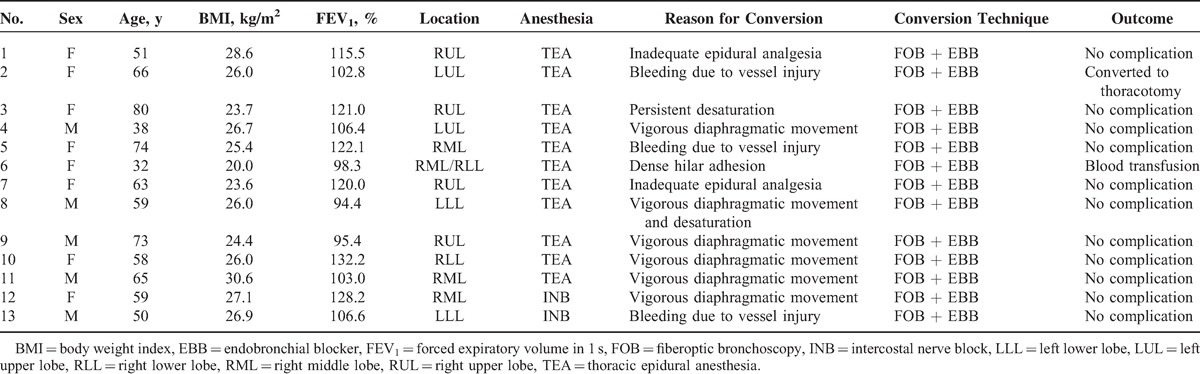
Characteristics of the Patients Converted to Tracheal Intubation and General Anesthesia

In patients that underwent INB, persistent hypotension that required medication and fluid replacement occurred significantly less often than in patients undergoing TEA. The mean blood loss of patients with INB was also significantly lower than in patients undergoing TEA.

### Postoperative Results

The TEA and INB groups had comparable pathological stages and numbers of dissected lymph nodes (Table 4). Postoperative complications developed in 26 patients (20.0%) among those receiving TEA, whereas 18 patients (16.7%) experienced postoperative complications in the INB group (*P* = 0.510). These complications included vomiting requiring medication, new onset of neurological deficits, prolonged air leak, subcutaneous emphysema, pneumonia, postoperative bleeding, and cardiac dysrhythmia (Table 4). Postoperative pain control was satisfactory in both the groups. The INB group had a shorter period of postoperative chest drainage and postoperative hospital stay in comparison with the TEA group (Table 4). There was no mortality within 30 days after surgery. One patient in the TEA group, unfortunately, developed acute transverse myelitis 2 days after surgery with permanent neurological deficits below T2.^20^

**TABLE 4:**
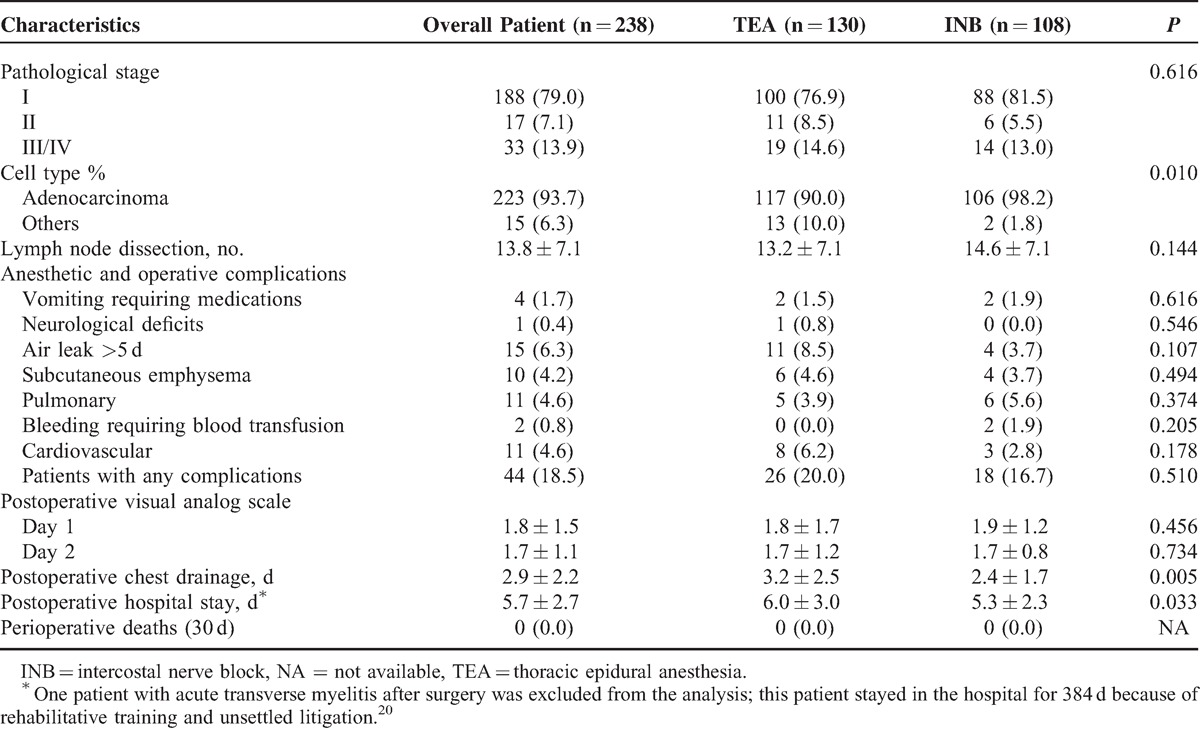
Postoperative Results by Group

### Multiple Regression Analyses

To evaluate the actual benefits of regional anesthesia with INB, multiple regression analyses were performed while adjusting for potentially confounding variables, including sex, history of smoking, preoperative lung function, and the ASA classification (Table 5). Patients in the INB group had significantly shorter anesthesia induction time and surgical duration. During surgery, INB was associated with better hemodynamic stability and required less fluid supplementation (mean difference: −782.8 mL; 95% CI, −948.8 to −616.8; *P* < 0.001), fewer vasoactive drugs (odds ratio: 0.53; 95% CI, 0.27 to 1.04; *P* = 0.064), and less blood loss (mean difference: −55.2 mL; 95% CI, −93.1 to −17.3; *P* = 0.004). After surgery, INB was associated with shorter duration of chest drainage (mean differences: −0.6 days; 95% CI, −1.2 to 0.0; *P* = 0.064) and similar length of hospital stay (mean differences: −2.0 days; 95% CI, −8.9 to 4.9; *P* = 0.569).

**TABLE 5:**
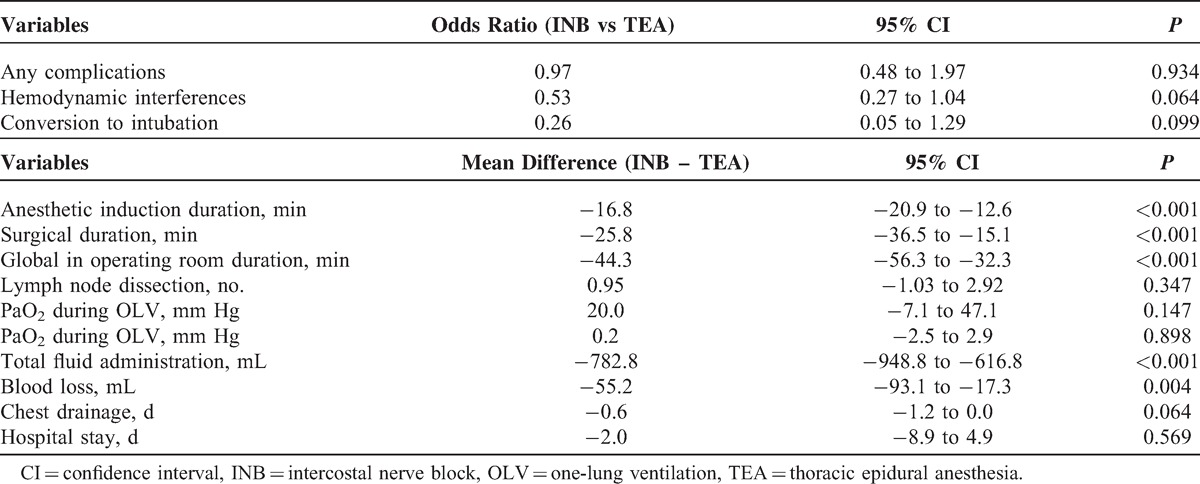
Multiple Regression Analysis and Comparisons of Perioperative Results Between the TEA and INB Groups

## DISCUSSION

The study results show that nonintubated thoracoscopic lobectomy using regional INB and TEA is safe and effective for patients with lung cancer. Additionally, INB is simpler and faster than TEA and interferes less with intraoperative hemodynamics. Thus, it requires less intraoperative fluid replacement.

Lobectomy is currently considered the standard of treatment for early-stage NSCLC.^2^ Recently, thoracoscopic surgery is becoming the preferred method to reduce postoperative pain, shorten hospital stay, and facilitate a fast recovery.^21^ As the use of minimally invasive surgical techniques evolves, surgeons and anesthesiologists expect less-invasive and equally effective anesthesia alternatives to evolve as well. Our initial studies using TEA in thoracoscopic lobectomy for NSCLC assured us that the use of regional anesthesia without intubation was not only feasible but also as safe as general anesthesia via intubation. In addition, it offered advantages including a trend toward better noncomplication rates (90% vs 66.7%, *P* = 0.057) and shorter hospital stays (mean, 5.9 vs 7.1 days, *P* = 0.078), not to mention prevention of intubation-related adverse effects.^10,11^ In spite of these encouraging results, our patients frequently hesitated to undergo surgery over fear of the use of neuroaxial anesthesia. Their concern stems from a cultural misconception that needle punctures around vertebral bones results in long-term back pain or even paralysis.^22^ Unfortunately, one 63-year-old man developed acute transverse myelitis 2 days after nonintubated thoracoscopic lobectomy with regional TEA.^20^ Although we excluded direct trauma due to the epidural catheterization and neurotoxicity from the local anesthetics used, this coincidental neurological event caused the patient permanent sensory loss below T2 and motor weakness of his lower extremities, over which he sued. Therefore, we sought an alternative regional anesthesia method other than thoracic epidural catheterization.

To our satisfaction, intercostal nerves can be easily located under direct thoracoscopic vision.^17^ The minimal effective dosage of local anesthetic can then be injected beneath the parietal pleura and along the intercostal nerves without the risk of needle-induced nerve injury or accidental intravenous injection of local anesthetics. Technically, the simplicity and accuracy of INB enable us to induce anesthesia faster without the need of time-consuming thoracic epidural catheterization and consequently make more efficient use of operating rooms. Physiologically, unlike TEA, which commonly produces a significant sympathetic block resulting in hypotension, INB induces less intraoperative hypotension. This significantly reduces the need for fluid replacement (*P* < 0.001) and vasoactive drugs (*P* < 0.001) during surgery. Although the role of fluid restriction in major pulmonary resections remains controversial,^23^ we believe that avoiding excess fluid administration reduces fluid overload, pulmonary edema, and impairment of gas exchange. Subsequently, there is less of a need to optimize respiratory function.^24^ Furthermore, the postoperative pain scores and the complication rates were comparable and satisfactory with both TEA and INB methods. By using INB, we found increased popularity for anesthetic methods without intubation among our patients undergoing thoracoscopic surgery. Thus, similar to the experience in the United Kingdom,^25^ use of TEA rapidly declined and is no longer regarded as the gold standard for acute pain management after thoracic surgery.^26,27^

In this cohort, most of the patients completed surgery with adequate sampling of mediastinal lymph nodes, except for the 13 patients (6.3%) who required conversion to tracheal intubation and general anesthesia. The need for conversion to tracheal intubation was due primarily to significant mediastinal movement (7 patients) jeopardizing a quiet and safe surgical environment, especially during dissection of the bronchovascular structures. In our experience, patients requiring conversion to tracheal intubation were mostly overweight with a BMI >26 kg/m^2^. Obesity decreases the functional residual capacity of the lung volume and increases the risk of hypoxemia during anesthesia. Furthermore, a distended diaphragm, especially the hemidiaphragm below the nonoperated lung, usually contracts more efficiently and exaggerates the mediastinal movement in spontaneously breathing patients with an open pneumothorax.^28^ We suggest that obese patients are not ideal candidates for thoracoscopic procedures without intubation. Consequently, a standby conversion plan should be prepared in advance and performed decisively in cases of large and uncontrollable amplitude of diaphragmatic motion. Tracheal intubation for one-lung isolation in a lateral position may be technically demanding with inherent risks, even by experienced thoracic anesthesiologists. It is our practice to intubate in the lateral position by aid of fiberoptic bronchoscopy, followed by inserting an endobronchial blocker to occlude the operated lung when tracheal intubation conversion indicated (Table 3). In difficult cases, the patients can also be deposited back to a supine position for tracheal intubation in a usual manner after inserting a temporary chest tube to reexpand the collapsed lung with wound covering.^10^

Although our patients experienced sedation-induced hypoventilation and paradoxical carbon dioxide rebreathing during OLV after creation of an iatrogenic open pneumothorax, they tolerated permissive hypercapnia well, with satisfactory arterial oxygen saturation via a facemask.

Despite of significantly shorter anesthetic and surgical durations, lower conversion rate, less blood loss, and shorter chest drainage in patients using INB, even so after multiple regression analyses of potentially preoperative confounding factors, these results should be interpreted cautiously. With 3 years of accumulated experience performing VATS without intubation under TEA, we shifted to INB for surgery without intubation in November 2012. As the learning curve progressed, we found that overweight patients (BMI > 26 kg/m^2^) were more likely to have vigorous diaphragmatic breathing, and we excluded such patients later in the study. Thus, progress in our patient selection and management methods for nonintubated VATS may partly explain the better results for nonintubated VATS using INB.

Our findings showed that there is no substantial difference in postoperative analgesia with INB when compared with TEA. Meanwhile, the surgical outcomes, complication rates, and length of hospital stay were comparable between the 2 surgical methods without intubation. We believe that using INB extends the indication of nonintubated VATS to a wider patient population, particularly to those patients in whom epidural catheterization is contraindicated.^17^ These contraindications include coagulopathy, local sepsis, preexisting neurologic disease, or spinal abnormalities.^17^ If anesthesiologists and patients are concerned about spinal cord injury after thoracic epidural techniques, INB may be easily and safely performed by surgeons without potential risk of spinal cord injury.^17^ For nonintubated VATS, the advantages and disadvantages of using TEA or INB are provided in Table 6.

**TABLE 6:**
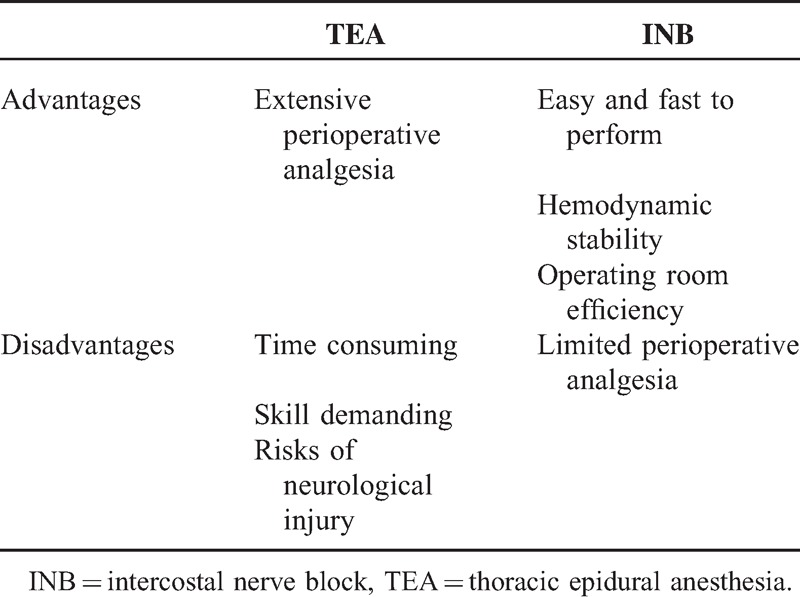
Advantages and Disadvantages of Thoracoscopic Lobectomy Without Intubation Using TEA or INB

Although this study was retrospective and the comparison was based on historical control patients in the cohort who did not undergo intubation, our overall experience shows that nonintubated thoracoscopic lobectomy may be performed safely and effectively in selected patients with early-stage NSCLC. INB is a less-invasive anesthetic alternative to TEA. INB is more efficient and promising for nonintubated VATS for the treatment of lung cancer. Further investigation is needed to clarify the long-term benefits of nonintubated thoracoscopic lobectomy using regional anesthesia from the perspective of oncological outomes and chronic pain compared with traditional general anesthesia requiring intubation.

## CONCLUSION

Nonintubated thoracoscopic lobectomy using either TEA or INB proved safe and technically feasible in selected patients with lung cancer. INB is a valuable, less-invasive technique that is as equally effective as TEA, but with the advantages of faster anesthetic induction and less hemodynamic interference during surgery. Although the long-term outcomes and oncological benefits require clarification in future, prospective randomized trials, we believe that nonintubated thoracoscopic lobectomy using INB is a simple and valid alternative to TEA for surgically managing selected patients with lung cancer.

## Acknowledgments

Statistical assistance was provided by the Taiwan Clinical Trial Bioinformatics and Statistical Center, Training Center, and Pharmacogenomics Laboratory, founded by the National Research Program for Biopharmaceuticals at the Ministry of Science and Technology of Taiwan (MOST 103-2325-B-002-033) and the Department of Medical Research at National Taiwan University Hospital, Taipei, Taiwan.
